# Clinical features and predictors of mortality among hospitalized patients with COVID-19 in Niger

**DOI:** 10.1186/s13031-021-00426-w

**Published:** 2021-12-14

**Authors:** Patrick D. M. C. Katoto, Issoufou Aboubacar, Batouré Oumarou, Eric Adehossi, Blanche-Philomene Melanga Anya, Aida Mounkaila, Adamou Moustapha, El khalef Ishagh, Gbaguidi Aichatou Diawara, Biey Joseph Nsiari-Muzeyi, Tambwe Didier, Charles Shey Wiysonge

**Affiliations:** 1 Cochrane South Africa, South African Medical Research Council, Francie van Zijl Drive, Parow Valley, Cape Town, 7501 South Africa; 2 Department of Global Health, Faculty of Medicine and Health Sciences, Stellenbosch University, Francie van Zijl Drive, Tygerberg, Cape Town, 7505 South Africa; 3 Centre for Infectious Diseases, Faculty of Medicine and Health Sciences, Stellenbosch University, Francie van Zijl Drive, Tygerberg, Cape Town, 7505 South Africa; 4 Centre for Tropical Medicine and Global Health, Faculty of Medicine, Catholic University of Bukavu, Bugabo 02, Bukavu, Democratic Republic of Congo; 5 Country Office, World Health Organization, Quartier Plateau, Avenue Mohamed VI, 1204 Niamey, Niger; 6 Department of Internal Medicine, Niamey General Reference Hospital, BP 12674, Niamey, Niger; 7 Directorate of Statistics, Ministry of Public Health, Niamey, Niger; 8 Direction of Surveillance and Response to Epidemics, Ministry of Public Health, Niamey, Niger; 9 Sub-Regional Office for West Africa, World Health Organization, Independence Street, Gate 0058, Ouagadougou, Burkina Faso; 10 School of Public Health and Family Medicine, University of Cape Town, Anzio Road, Observatory, Cape Town, 7935 South Africa

**Keywords:** Humanitarian crisis, SARS-CoV-2, Baseline characteristics, Mortality, Africa

## Abstract

**Introduction:**

COVID-19 has spread across the African continent, including Niger. Yet very little is known about the phenotype of people who tested positive for COVID-19. In this humanitarian crises region, we aimed at characterizing variation in clinical features among hospitalized patients with COVID-19-like syndrome and to determine predictors associated with COVID-19 mortality among those with confirmed COVID-19.

**Methods:**

The study was a retrospective nationwide cohort of hospitalized patients isolated for COVID-19 infection, using the health data of the National Health Information System from 19 March 2020 (onset of the pandemic) to 17 November 2020. All hospitalized patients with COVID-19-like syndrome at admission were included. A Cox-proportional regression model was built to identify predictors of in-hospital death among patients with confirmed COVID-19.

**Results:**

Sixty-five percent (472/729) of patients hospitalized with COVID-19 like syndrome tested positive for SARS-CoV-2 among which, 70 (15%) died. Among the patients with confirmed COVID-19 infection, age was significantly associated with increased odds of reporting cough (adjusted odds ratio [aOR] 1.02; 95% confidence interval [CI] 1.01–1.03) and fever/chills (aOR 1.02; 95% CI 1.02–1.04). Comorbidity was associated with increased odds of presenting with cough (aOR 1.59; 95% CI 1.03–2.45) and shortness of breath (aOR 2.03; 95% CI 1.27–3.26) at admission. In addition, comorbidity (adjusted hazards ratio [aHR] 2.04; 95% CI 2.38–6.35), shortness of breath at baseline (aHR 2.04; 95% CI 2.38–6.35) and being 60 years or older (aHR 5.34; 95% CI 3.25–8.75) increased the risk of COVID-19 mortality two to five folds.

**Conclusion:**

Comorbidity, shortness of breath on admission, and being aged 60 years or older are associated with a higher risk of death among patients hospitalized with COVID-19 in a humanitarian crisis setting. While robust prospective data are needed to guide evidence, our data might aid intensive care resource allocation in Niger.

## Introduction

The novel coronavirus disease 2019 (COVID-19), a disease caused by severe acute respiratory syndrome coronavirus 2 (SARS-CoV-2) has been declared a pandemic by the World Health Organization due to both the unprecedented levels of transmission and the magnitude of the epidemic [1]. As of 25 November 2020, 47 African countries were impacted with 1,451,296 total cases of COVID-19and 24,454 deaths reported [2]. Niger, one of the West African nations, announced its first case of COVID-19 in the capital city, Niamey, on 19 March 2020. As of 25 November 2020, there were 1,381 confirmed cases of the virus, including 70 deaths [3]. The pandemic in Niger, a Lake Chad region, is superimposed over various dynamics of insecurity and states of emergency due to constant attacks by armed groups, now lasting more than a decade [4]. The scale of this state of violence has been characterized by an ongoing humanitarian crisis in some areas, such as the Diffa region [5] that has weakened the health system in the country. Several health services have closed, and others have been overwhelmed by the high demand from locals as well as displaced and immigrant populations.

Although the literature on COVID-19 is growing at an exponential pace, there is still a shortage of observational studies from several African countries. However, local epidemiology is essential to enable the adaptation of the evidence-based response of COVID-19 at local levels as well as national and regional levels, once pooled [6, 7]. For example, in typical resource constrained settings, not every facility would have adequate intensive care beds, access to ventilators, continuous oxygen, CT scanners, or even basic biological testing kits. Thus, characterizing admitted patients who require an intensive care unit (ICI) bed, and using these characteristics in a risk assessment, can help to manage current resources, and plan for future needs [8].

In this particular context of Niger, we aimed to describe the clinical characteristic of patients hospitalized with COVID-19 like syndrome to compare the variation in clinical features among all hospital patients quarantined for COVID-19 infection and determine independent factors associated with COVID-19 mortality among the patients with confirmed COVID-19, to help inform triage decisions and proper allocation of medical resources.

## Methods

### Study design, setting and population

We conducted a retrospective cohort study in Niger among hospitalized patients quarantined for COVID-19 infection; from 19 March 2020 (onset of the pandemic) to 17 November 2020. Niger is a large nation in the heart of the Sahel region with a growing population estimated at 24,811,942 in 2020 [9]. Despite major measures taken over the last decade by the government of Niger to reduce poverty in the country, the severe poverty rate had remained considerable at 41.4% in 2019, impacting more than 9.5 million people. In recent years, Niger has also faced a major influx of refugees fleeing war in adjacent regions, especially from Nigeria and Mali (221,671 refugees and 196,717 displaced people, primarily in Diffa and Maradi in 2019) [10]. In Niger, the health sector is structured in three tiers: the central level determining the general plan and running national hospitals and health centers; the regional level, including the eight Directions Generales de la Santé Publique (DRSP), defined by the six regional hospitals and two comparison centers; and the third level includes 42 Equipes Cadres du District (ECD) in 42 district hospitals and the associated network of 578 centre de santé intégrés and 1201 aires de santé. All symptomatic COVID-19 patients are managed at level 1 and 2. In our study, we included all patients from all eight political regions (Niamey, Agadez, Diffa, Dosso, Maradi, Tahaoua, Tillaberi and Zinder) hospitalized in level 1 and 2 regardless of age or nationality, and whose health data were available in the National Health Information System (DHIS2). This information was supplemented as needed by consultation registers. All outpatients were excluded regardless of COVID-19 results. Hospitalized patients with missing data on the outcome of interest were also excluded.

### Data collection procedures and variables definition

From the health data of the DHIS2, we collected explanatory variables in a Microsoft Excel sheet. These variables included socio-demographic data (age, sex, profession), travel information during the past 14 days, contact with a patient with confirmed COVID-19, history and type of comorbidity (cardiovascular and hypertension, obesity, diabetes, HIV-infection, tuberculosis, asthma and other chronic respiratory diseases, chronic kidney diseases and any type of cancer), symptoms at admission (fever/chills, sore throat, cough, rhinorrhea, shortness of breath, digestive signs, headache, asthenia/ fatigue, musculoskeletal pain and thoracic pain) as well as the time between first symptoms occurrence and the first visit at a recognized health center. We collected and used the result of SARS-CoV-2 RT-PCR testing to to distinguish between positive and negative isolated hospitalized patients with suspected COVID-19 infection. One dependent variable, hospitalization outcome (alive or dead or unknown), was also collected.

### Data analysis

We described the data in terms of frequencies and percentages, means and standard deviations (SDs), or medians and interquartile ranges (IQRs). Chi-square (exact), t-tests and Wilcoxon rank sum tests were applied to test for associations, where applicable. To identify independent predictors associated with the risk of experiencing specific respiratory symptoms (cough, sore throat, shortness of breath, thoracic pain, and rhinorrhea) at admission, we built a multivariable logistic regression, using a backward stepwise approach, model. This model was complemented by manual selection of a priori variables of clinical or epidemiological relevance. The likelihood of overall survival between age groups was determined using the Kaplan–Meier method. We used univariate and multivariate Cox-proportional regression modeling to identify predictors independently associated with the odds of mortality among hospitalized patients with confirmed COVID-19 infection. The final model contained age (as continuous), sex and other predictors of significance (*p* < 0.05), defined by a stepwise model method. The variables included in the final model were checked for proportionality assumptions and no first-order interactions were found. The strength of the relationship was represented as adjusted odds ratios (aOR) for multivariate logistic regression or adjusted hazard ratio (aHR) for Cox-proportional regression and corresponding 95% confidence intervals (CI). Reported p-values are exact and two-tailed, and a value < 0.05 was considered statistically significant. Stata software version 14.1 (Stata, College Station, TX) was used for all data analysis.

## Results

### Characteristics of patients hospitalized for suspected COVID-19 infection

**Fig. 1:**
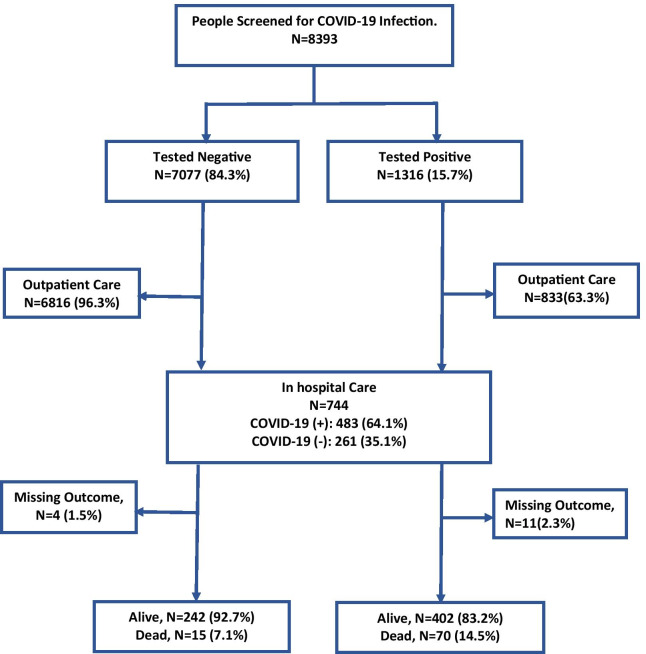
Flow chat of the study

Of the 8393 persons screened during the study period, 1316 (16%) tested positive for COVID-19. Of the 729 hospitalized patients with COVID-19-like syndrome, 472 (65%) tested positive for the disease. Of these, 70 (15%) died with SARS-CoV-2 infection (Fig. 1). The details of the characteristics of the patients can be found in Table 1. Patients hospitalized for a suspected COVID-19 infection who remained negative for SARS-CoV-2 were significantly younger (median and interquartile range (IQR), 40 (27–60) years) compared to those who tested positive for SARS-CoV-2 but remained alive (median (IQR) 45 (29–59) years) and those who died (median (IQR) 65 (29–59) years). No such differences were observed in gender-based analysis, but patients hospitalized for suspected COVID-19 infection who tested positive were more likely to be working in private sectors. While only 120/729 (16%) of hospitalized patients with suspected COVID-19 indicated recent international travel within 14 days prior, 62/729 (13%) of these patients were quarantined (with suspected infection) at entry points and 125/729 (18%) were in contact with a person with confirmed COVID-19 infection. Among this cohort, a history of comorbidity was common (31%) and more prevalent among those who tested positive for COVID-19, or those who died; with cardiometabolic diseases and chronic respiratory illnesses being the most frequently reported. Overall, the time between showing first symptoms and the first hospital visit was shorter in patients who tested positive for COVID-19 than those who tested negative. The most frequent symptoms reported were cough (176/729; 24%), fever/chills (165/729; 23%) and shortness of breath (118/729; 16%). Half of patients who died had presented with fever/chills and/or shortness of breath; the mean time to death was just under five days.

**Table 1:** Demographic and clinical characteristics of 729 patients hospitalized for COVID-19 suspicion in Niger

Variables	Alive COVID-19 (−) N = 242 (33.20%)	Alive COVID-19 (+) N = 402 (55.14%)	Dead N = 85 (11.66%)	All N = 729	*P*-values
Age (median/IQR)	40 (26.5–60)	45 (29–59)	65 (29–59)	46 (30–61)	0.0001
*Age groups*					
< 15 years	16 (6.67)	8 (2.00)	2 (2.35)	26 (3.58)	< 0.0001
15–34 years	87 (36.25)	125 (31.17)	5 (5.88)	217 (29.89)	
35–59 years	71 (29.58)	174 (43.39)	23 (27.06)	268 (36.91)	
≥ 60 years	66 (27.50)	94 (23.44)	55 (64.71)	215 (29.61)	
*Sex*					
Male	171 (70.66)	296 (73.63)	57 (67.06)	524 (71.88)	0.401
*Profession*					
Student	7 (7.45)	41 (10.93)	0 (0.00)	48 (8.76)	< 0.0001
Public Servant/Administration	6 (6.38)	89 (23.73)	23 (29.11)	118 (13.69)	
Housekeeper/none	25 (26.60)	30 (8.00)	20 (25.32)	75 (13.69)	
Private	48 (51.06)	150 (40.00)	34 (43.04)	232 (42.34)	
Health workers	8 (8.51)	65 (17.33)	2 (2.53)	75 (13.69)	
*Travel last 14 days*					
Yes	24 (10.57)	91 (22.64)	5 (5.88)	120 (16.81)	< 0.0001
*Detected at entry port*					
Yes	0 (0.00)	62 (15.42)	0 (0.00)	62 (12.73)	< 0.0001
*Contact of confirmed case*					
Yes	5 (2.21)	107 (26.62)	13 (15.48)	125 (17.56)	< 0.0001
*Visited hospital last 14 days*					
Yes	40 (18.43)	83 (20.65)	19 (22.62)	142 (20.20)	0.66
*History of comorbidity*					
Chronic Respiratory Diseases	8 (47.06)	14 (63.64)	5 (71.43)	27 (58.70)	0.44
CVD/HPT	12 (57.14)	52 (86.67)	32 (94.12)	96 (83.48)	0.001
Diabetes	9 (50.00)	29 (78.38)	20 (90.91)	58 (75.32)	0.01
Obesity	0 (0.00)	0 (0.00)	2 (50.00)	2 (9.52)	0.029
HIV (+)	0 (0.00)	3 (27.27)	0 (0.00)	3 (13.64)	0.29
Tuberculosis	2 (18.18)	1 (11.1)	0 (0.00)	3 (13.64)	0.76
CKD	4 (30.77)	0 (0.00)	1 (33.33)	5 (20.83)	0.21
Any cancer	0 (0.00)	2 (20.00)	2 (50.00)	4 (17.39)	0.1
Any comorbidity	46 (21.10)	119 (29.60)	53 (63.10)	218 (30.97)	< 0.0001
≥ 2 Comorbidities	6 (40.00)	25 (75.76)	19 (90.48)	50 (72.46)	0.004
Time symptoms-consultation (mean ± SD)	3.5 (5.81)	2.42 (4.23)	2.69 (3.51)	2.76 (4.65)	< 0.0001
*Symptoms*					
Fever/chills	4 (1.65)	118 (29.35)	43 (50.59)	165 (22.63)	< 0.0001
Sore throat	3 (1.24)	55 (13.68)	7 (8.24)	65 (8.29)	< 0.0001
Cough	4 (1.65)	136 (33.83)	36 (42.35)	176 (24.14)	< 0.0001
Rhinorrhea	0 (0.00)	38 (9.45)	7 (8.24)	45 (6.17)	< 0.0001
Shortness of breath	2 (0.83)	73 (18.16)	43 (50.59)	118 (16.19)	< 0.0001
Anosmia/Ageusia	7 (5.79)	6 (4.03)	3 (8.33)	16 (5.23)	0.5
Thoracic pain	35 (28.93)	27 (18.24)	5 (13.89)	67 (21.97)	0.05
Digestive signs	30 (24.79)	32 (21.62)	7 (19.44)	69 (22.62)	0.73
Headache	38 (31.40)	36 (24.32)	6 (16.67)	80 (26.23)	0.16
Asthenia/fatigue	35 (28.93)	25 (16.89)	8 (22.22)	68 (22.30)	0.06
Musculoskeletal pain	7 (5.79)	13 (8.78)	2 (5.56)	22 (7.21)	0.59
Time hospitalization-discharge/death (mean ± SD)	12 (0.10)	14.31 (6.61)	4.74 (5.11)	12.6 (7.33)	< 0.0001

All values are count and percentage unless otherwise indicated

SD, standard deviation; IQR, interquartile range; CVD/HPT, cardiovascular diseases/hypertension; CKD, chronic kidney diseases; HIV, human immunodeficiency virus

### Predictors of respiratory symptoms in in-patients with COVID-19 infection

Table 2 displays predictors of respiratory symptoms among hospitalized patients with confirmed SARS-CoV-2 infection. Every increase in year of age was significantly associated with increased odds of reporting cough (aOR 1.02; 95% CI 1.01–1.03), fever/chills (aOR 1.02; 95% CI 1.02–1.04) but was also associated with a slightly reduced in risk of reporting rhinorrhea (aOR 0.97; 95% CI 0.95–0.99). Men were more likely to report cough (aOR 1.55; 95% CI 1.0–2.50) and thoracic pain (aOR 2.26; 95% CI 0.90–5.65) compared to women but the difference was not statistically significant for thoracic pain. Patients with any history of comorbidity had 1.59 (95% CI 1.03–2.45) and 2.03 (95% CI: 1.27–3.26) higher odds of presenting cough and shortness of breath on admission, respectively. Every day delayed between the first symptom and first hospital visit was independently associated with higher odds of presenting fever/chills at admission (aOR 1.06; 95% CI 1.01–1.11).

**Table 2:** Odds ratios (95% confidence interval) for respiratory symptom outcomes estimated by multivariable logistic regression among 472 patients hospitalized for confirmed COVID-19 infection

Variables	Cough	Sore throat	Fever/chills	Shortness of breath	Thoracic pain	Rhinorrhea
	aOR (95% CI); *p*-value	aOR (95% CI); *p*-value	aOR (95% CI); *p*-value	aOR (95% CI); *p*-value	aOR (95% CI); *p*-value	aOR (95% CI); *p*-value
Age (years)	1.02 (1.01–1.03); 0.001	0.99 (0.98–1.01); 0.48	1.02 (1.01–1.03); < 0.0001	1.03 (1.02–1.04); < 0001	1.00 (0.98–1.02); 0.86	0.97 (0.95–0.99); 0.005
*Age-group* (*years)*						
< 15	Ref	Ref	Ref	Ref	Ref	Ref
15–34	0.88 (0.21–3.63); 0.86	0.53 (0.10–2.74); 0.45	0.69 (0.17–2.84); 0.61	0.33 (0.08–1.44); 0.14	NE	2.15 (0.22–20.70; 0.51
35–59	1.27 (0.31–5.09); 0.74	0.69 (0.14–3.44); 0.65	1.28 (0.32–5.11); 0.73	0.51 (0.13–2.09); 0.35	1.29 (0.41–3.98); 0.66	3.51 (0.23–54.25); 0.37
> 60	1.90 (0.47–7.66); 0.37	0.48 (0.09–2.47); 0.38	1.54 (0.38–6.20); 0.54	1.56 (0.38–6.27); 0.53	1.47 (0.61–3.55); 0.38	7.20 (0.23–227.3); 0.26
*Sex*						
Female	Ref	Ref	Ref	Ref	Ref	Ref
Male	1.55 (1.0–2.40); 0.05	1.10 (0.60–2.03); 0.86	1.29 (0.82–2.05); 0.27	0.77 (0.48–1.23); 0.27	2.26 (0.90–5.65); 0.08	0.94 (0.47–1.85); 0.85
*Profession*						
Pupil/student	Ref	Ref	Ref	Ref	Ref	Ref
Public Servant/Administration	1.31 (0.52–3.30);0.56	2.51 (0.67–9.35); 0.17	0.77 (0.30–1.94); 0.58	1.17 (0.39–3.52); 0.77	1.15 (0.10–12.86); 0.91	8.82 (1.66–46.91); 0.01
Housekeeper/None	0.61 (0.19–1.9); 0.40	1.24 (0.25–6.13); 0.79	0.56 (0.19–1.69); 0.31	0.91 (0.26–3.18); 0.88	4.89 (0.34–70.78); 0.24	3.23 (0.46–23.35); 0.25
Private	1.22 (0.51–2.92); 0.65	2.36 (0.68–8.10); 0.17	0.74 (0.31–1.75); 0.50	0.89 (0.31–2.58); 0.83	2.31 (0.22–23.82); 0.48	2.69 (0.52–13.89); 0.24
Health workers	1.18 (0.48–2.97); 0.71	1.94 (0.53–7.09); 0.32	0.76 (0.31–1.92); 0.49	0.66 (0.21–2.04); 0.47	0.63 (0.05–8.83); 0.73	5.49 (1.07–28.21); 0.04
*Travel last 14 days*						
No	Ref	Ref	Ref	Ref	Ref	Ref
Yes	0.34 (0.21–0.66); 0.001	1.12 (0.578–2.18); 0.74	0.44 (0.25–0.79); 0.006	0.42 (0.21–0.85); 0.017	1.06 (0.21–5.33); 0.94	0.21 (0.06–0.73); 0.013
*Any Comorbidity*						
No	Ref	Ref	Ref	Ref	Ref	Ref
Yes	1.59 (1.03–2.45); 0.04	0.85 (0.45–1.61); 0.62	1.49 (0.96–2.30); 0.08	2.03 (1.27–3.26); 0.003	0.80 (0.35–1.85); 0.61	1.07 (0.51–2.21); 0.87
≥ *2 Comorbidities*						
No	Ref	Ref	Ref	Ref	Ref	Ref
Yes	1.18 (0.90–1.55); 0.21	NE	1.19 (0.91–1.54); 0.20	1.09 (0.84–1.41); 0.50	NE	NE
Time symptoms-consultation	1.04 (0.99–1.09); 0.09	0.99 (0.92–1.07); 0.84	1.06 (1.01–1.11); 0.02	1.04 (0.98–1.09); 0.20	1.05 (0.96–1.15); 0.23	1.02 (0.95–1.11); 0.55

All models are adjusted by age and sex

aOR, adjusted odds ratio; Ref, reference; NE, not estimable

### Predictors of mortality in in-patients with COVID-19 infection

**Fig. 2:**
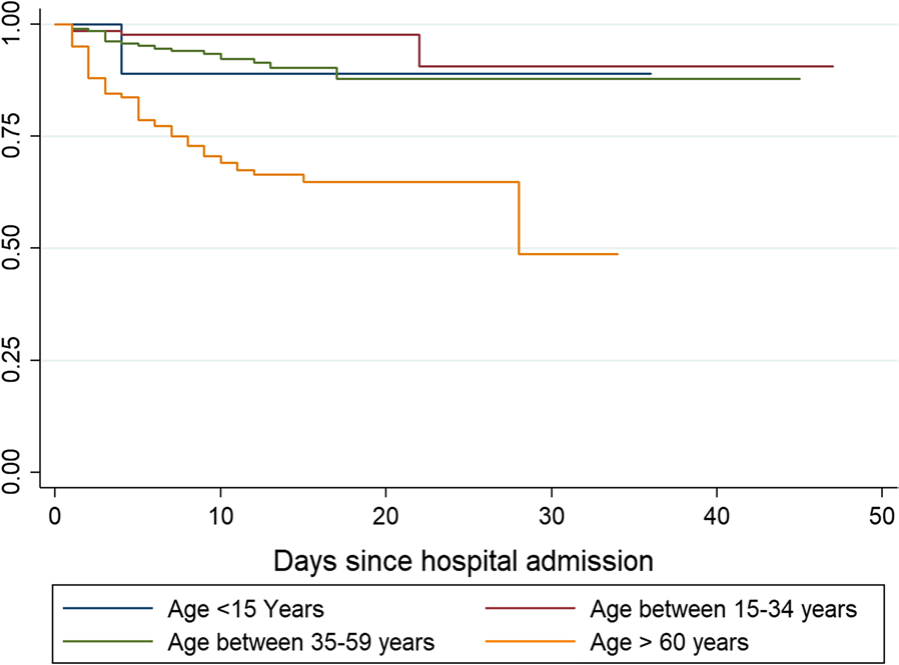
Kaplan–Meier curves displaying the estimated survival time stratified by age of hospitalized patients with laboratory confirmed COVID-19 infection in Niger from March 2020 to November 2020. *Note*: Days since hospital admission is extended to 45 days corresponding to the longest hospitalization. The steps in the graph correspond to patient’s death-points. Discharged patients were censored at time of discharge

Table 3 and Fig. 2 depict predictors associated with death among hospitalized patients with confirmed COVID-19 infection. After holding constant other variables in the model, every one-year increase in age was independently associated with the hazard of death (aHR 1.05; 95% CI 1.03–1.08) among hospitalized patients with COVID-19 infection. In addition, compared to patients aged below 60 years, patients aged 60 years or above exhibited a more than fivefold increase in the hazard of death (aHR 5.34; 95% CI 3.25–8.75). No sex difference was observed in the hazards of death between males and females. The hazard of death among hospitalized patients with SARS-CoV-2 infection and a history of any comorbidity was more than double than that for those without history of any comorbidity (aHR 2.04; 95% CI 2.38–6.35). Similarly, patients admitted with baseline shortness of breath had significantly higher hazard of death than patients without baseline shortness of breath at admission (aHR 2.09; 95% CI 1.22–3.57).

**Table 3:** Univariable and multiple cox regression analysis of predictors associated with death among 472 patients hospitalized for confirmed COVID-19 infection

Variables	Crude HR (95% CI)	*P*-value	Adj. HR (95% CI)	*P*-value
Age	1.06 (1.04–1.08)	< 0.0001	1.05 (1.03–1.07)	< 0.0001
*Age group 1*				
< 15 years	Ref	Ref	_	_
15–34 years	0.31 (0.03–2.82)	0.30	_	_
35–59 years	0.89 (0.12–6.83)	0.91	_	_
> 60 years	3.76 (0.51–27.88)	0.19	_	_
*Age-group 2*				
< 60 years	Ref	Ref	Ref	Ref
> 60 years	5.34 (3.25–8.75)	< 0.0001	3.32 (1.88–5.89)	< 0.0001
*Sex*				
Female	Ref	Ref	Ref	Ref
Male	0.80 (0.48–1.31)	0.37	0.85 (0.50–1.45)	0.56
*Profession*				
Student/housekeeper/none	Ref	Ref	_	_
Other	0.76 (0.43–1.34)	0.34	_	_
*Travel last 14 days*				
No	Ref	Ref	Ref	Ref
Yes	0.29 (0.12–0.72)	0.007	0.77 (0.32–1.87)	0.57
*Visited hospital last 14 days*				
No	Ref	Ref	_	_
Yes	1.13 (0.65–1.96)	0.65	_	_
History of comorbidity				
*Any comorbidity*				
No	Ref	Ref	Ref	Ref
Yes	3.89 (2.38–6.35)	< 0.0001	2.04 (1.22–3.42)	0.006
* ≥ 2 Comorbidities*				
No	Ref	Ref	_	_
Yes	1.13 (0.91–1.39)	0.25	_	_
Time symptoms-consultation	1.00 (0.95–1.05)	0.98	_	_
Symptoms				
*Fever/chills*				
No	Ref	Ref	_	_
Yes	2.24 (1.41–3.56)	0.001	1.21 (0.74–1.99)	0.45
*Sore throat*				
No	Ref	Ref	_	_
Yes	0.62 (0.27–1.44)	0.27	_	_
*Cough*				
No	Ref	Ref	_	_
Yes	1.36 (0.85–2.16)	0.19	_	_
*Rhinorrhea*				
No	Ref	Ref	_	_
Yes	0.82 (0.36–1.90)	0.65	_	_
*Shortness of breath*				
No	Ref	Ref	Ref	Ref
Yes	3.94 (2.49–6.23)	< 0.0001	2.09 (1.22–3.57)	0.007
*Anosmia/Ageusia*				
No	Ref	Ref	_	_
Yes	1.07 (0.13–8.64)	0.95	_	_
Thoracic Pain				
No	Ref	Ref	_	_
Yes	0.69 (0.23–2.06)	0.51	_	_
*Digestive signs*				
No	Ref	Ref	_	_
Yes	0.56 (0.19–1.62)	0.28	_	_
*Headache*				
No	Ref	Ref	_	_
Yes	0.51 (0.18–1.43)	0.2	_	_
*Asthenia/fatigue*				
No	Ref	Ref	_	_
Yes	1.24 (0.51–2.99)	0.63	_	_

Adj. HR, adjusted hazards ratio; Ref, reference; _, indicates variables not included in the multivariate model

## Discussion

In this first retrospective cohort study from Niger, we report the phenotype of patients hospitalized for COVID-19 suspicion, predictors associated with the odds of respiratory symptoms and predictors associated with the hazards of death among those who tested positive for COVID-19. We found that predictors independently associated with in-hospital mortality in relation to COVID-19 infection were age (mainly 60 years or older), history of comorbidity and baseline shortness of breath.

Socio-demographic and clinical parameters linked to the severity and mortality of COVID-19 have been widely reviewed [11–15]. Age is considered to be strongly correlated with COVID-19 outcomes globally [14, 16], but little is known about the situation on the African continent. A recent systematic review (n = 15 studies) reported that elderly adults and people with comorbidities suffer severe forms of COVID-19 and are at increased risk of hospitalization and death [17]. We found that patients hospitalized with a suspected COVID-19 infection who remained negative for SARS-CoV-2 were significantly younger than those who tested positive for SARS-CoV-2, as well as those who died. In addition, we found that every increase in year of age, was significantly associated with higher odds of reporting cough or fever/chills but was associated with a slightly reduced in risk of reporting rhinorrhea. Humans have developed natural immunity and immunological memory to withstand repeated infections. However, dysregulated adaptative immunity associated with the ageing process is known to increase the risk of morbidity following a decline in the immune system [18]. For example, roughly 90% of the excess deaths for seasonal influenza happen in elderly people. There is progressive lymphopenia with CD4 + T-cell attrition in the aging immune system, and reduced regulatory T-cell activity that contribute to homeostatic proliferation of lymphocytes with autoimmune tendency and unnecessary inflammatory responses [19]. Lymphopenia is a hallmark of SARS-CoV-2 infection [20] and is affected by several variables such as those that impact survival. Infection, such as with COVID-19, then exacerbates the imbalanced aged immune system, thereby exacerbating the loss of CD4 + T cells and inflammatory macrophage reaction [19].

In agreement with our findings, a study of 1028 confirmed cases of COVID-19 from Africa has also identified chronic diseases as an independent factor associated with death among patients infected with SARS-CoV-2 [21]. Chronic conditions are often associated with a sub-clinical level of inflammation, weakened innate immune responses and a strong ACE-2 receptor facilitating SARS-CoV-2 entry into the host cells [11, 13]; and correlating with COVID-19 severity [22]. These comorbidities carry the COVID-19 patient through a vicious infectious life cycle (amplification of cytokine storm) and are significantly correlated with severe morbidity and mortality [13, 23]. In our cohort, a history of comorbidity was common and more prevalent among those who tested positive for COVID-19; cardiometabolic diseases (cardiovascular diseases, hypertension, and diabetes) and chronic respiratory illnesses (COPD and asthma) were the most frequently reported. This is in line with previous data [24] (participants, n = 202,005 patients with COVID-19) showing any type of chronic comorbidity, hypertension/cardiovascular disease, diabetes, and respiratory diseases were the most prevalent chronic comorbid conditions. As in our study, polymorbidity was also common. However, the extent to which comorbidities influence the pandemic remains questionable. Previous research synthesis have shown some methodological limitations by using preprint data and limited global clinical data [11, 25]. A more recent meta-analysis of published data from large cohorts from across the world reported that hypertension, diabetes, and cancer significantly exacerbate the severity of COVID-19 in patients resulting in death, however, chronic kidney diseases contributed the most to death [11]. Analyzing the association between chronic comorbidity and COVID-19 severity/fatality by country of residence, Zhou et al. found that such odds were highly and significantly increased for obesity in France (compared to that in USA, UK and China), for hypertension/ cardiovascular diseases in China and for diabetes in the USA [26]. HIV-infection and/or tuberculosis independently predicted the hazard of death in South Africa [12] and of severity in Ethiopia [27], but not in studies from the USA [15, 28]. As such, the call by The African Forum for Research and Education in Health [6, 7] to pool COVID-19 data from African countries very necessary to enhance COVID-19 epidemiology in Africa to ensure an adaptive response at both hospital and community levels.

COVID-19 typical symptoms include fever, cough and shortness of breath [29–32]. The most frequent symptoms reported in our cohort were cough, fever/chills and shortness of breath with half of patients who died exhibiting fever/chills and/ or shortness of breath at presentation. Furthermore, shortness of breath was also a predictor of death. Latest evidence indicate that a number of patients with serious COVID-19 might have the cytokine storm syndrome [23]. The cytokine storm syndrome might explain COVID-19-related clinical symptoms and signs such as fever, cough/sputum, shortness of breath, faster respiratory rates, stuffy nose and generalized malaise, as these are common to other diseases-associated cytokine storm syndrome [33]. However, shortness of breath (regardless of degree of severity) has been identified as critically important core outcomes by more than 9,300 patients, health professionals, and the public from 111 countries in the global Coronavirus Disease 2019 Core Outcome Set Initiative [34]. This is important since it will help to capture the dynamic nature of COVID-19, illustrate the debilitating severity, and measure the progress in the resumption of daily activities. The congestion of the intensive care unit can be correlated with the fatality of COVID-19 [35]. Prioritizing patients in need of intensive treatment is required to reduce the death rate during the pandemic [36]. Therefore, knowing the predictive potential of baseline symptoms in a deadly pandemic is particularly valuable in settings with resource shortages, such as rural Africa. In agreement with many prognosis scores simply excluding laboratory/imagery data (machine learning model or not), shortness of breath/ dyspnea significantly predicted COVID-19 severity (hospitalization, stay in intensive care and or death) [8, 35, 36]. Moreover, in one model, laboratory data only predicted clinical deterioration while baseline clinical data such age and shortness of breath/ dyspnea significantly predicted the hazards of death among critically ill patients [37].

Although informative, our research highlights the need to design prospective studies to address the identified shortfalls. The use of registry-based data designed at the onset of the pandemic (at risk of missing data) is one of the main shortcomings of our research. This did not allow us to provide a full understanding of comorbidity (e.g., very low prevalence of reported obesity and HIV) and, as a result, we did not include each comorbidity in the updated models to prevent misleading interpretation. Similarly, roughly 2% of patients hospitalized for COVID-19 missed final clinical outcome but no difference was observed for other baseline characteristics compared to included patients. Moreover, COVID-19 clinical severity, laboratory and imaging results were not routinely recorded in the national public health record and were consequently not included in this first study. Although one might assume that hospitalized patients were more likely to present with moderate to severe COVID-19 stage we also acknowledge that not considering this information might bias our findings either away, or towards the true effect. However, our research included a comprehensive data from all eight provinces in Niger and thus offered a thorough real-world description of COVID-19 epidemiology in this humanitarian setting. Moreover, while this is the first report from Niger, we are also the first to compare the variation in clinical features among all hospitalized patients quarantined for COVID-19 infection, considering this region has the highest burden of other respiratory diseases in Africa.

## Conclusion

In this retrospective study, respiratory distress upon admission, as measured by shortness of breath, high-risk age groups, and comorbidities significantly predicted mortality among COVID-19 patients hospitalized in Niger. In addition, by following the inpatient route during COVID-19 pandemic, we have characterized the clinical profile of patients needing extensive care in resource-constrained settings to assist risk-adapted choices on the allocation of medical resources. Further research may concentrate on converting existing data into a meaningful prognostic score for physicians working in conflict zones.

## Data Availability

The datasets used and/or analyzed during the current study are available from the corresponding author on reasonable request.

## Abbreviations

ACE-2: Angiotensin-converting enzyme 2
aHR: Adjusted hazards ratio
aOR: Adjusted odds ratio
CD4: Cluster of differentiation 4
COPD: Chronic obstructive pulmonary diseases
COVID-19: Novel coronavirus disease 2019
DHIS2: District health information software 2
DRSP: Directions générales de la santé publique
ECD: Equipes cadres du district
HIV: Human immunodéficience virus
RT-PCR: Real time polynuclear chain reaction
SARS-CoV-2: Severe acute respiratory syndrome coronavirus 2
UK: United Kingdom
USA: United State of America
